# Experiences With a Multicomponent Digital Behavioral Pain Management Intervention for Adults With Sickle Cell Disease: Qualitative Analysis of the CaRISMA Trial

**DOI:** 10.2196/73719

**Published:** 2025-08-05

**Authors:** Olubusola B Oluwole, Lakeya S McGill, Kadeem Gayle, Julia A O'Brien, Kaleab Z Abebe, Charles R Jonassaint, Megan Hamm

**Affiliations:** 1 Department of Medicine University of Pittsburgh Pittsburgh, PA United States; 2 Department of Acute and Tertiary Care School of Nursing University of Pittsburgh Pittsburgh, PA United States; 3 Center for Biostatistics & Qualitative Methodology Pittsburgh, PA United States

**Keywords:** behavioral, digital intervention, sickle cell disease, chronic pain, pain

## Abstract

**Background:**

Chronic pain is prevalent among adults with sickle cell disease (SCD) and can be worsened by psychosocial factors such as depression and inadequate social support. Effective behavioral interventions (eg, cognitive behavioral therapy [CBT]) exist for chronic pain in various populations; however, few have been developed to address chronic pain in SCD. Several barriers have restricted the development and dissemination of CBT pain interventions in SCD, such as limited accessibility and time constraints. Digital interventions provide accessible and cost-effective pain management tools, offering self-management strategies, real-time monitoring, and personalized treatment options. Yet, there are limited data regarding patients’ experiences with such interventions within the SCD population. The Cognitive Behavioral Therapy and Real-Time Pain Management Intervention for Sickle Cell Via Mobile Applications (CaRISMA) trial evaluated the effectiveness of a digital CBT intervention compared with a digital educational intervention for pain management in SCD. Evaluating participants’ experiences can guide refinement of digital pain interventions in SCD.

**Objective:**

This study aimed to gain a deeper understanding of the lived experiences of participants in the CaRISMA trial and to determine how to better adapt this intervention to the SCD population. The study examined individuals’ overall experience with the trial and their perspectives of the trial components: a health coach, a chatbot-delivered digital CBT program, and an electronic pain diary.

**Methods:**

Respondents were randomly selected to participate in semistructured interviews at (1) baseline, (2) the end of the intervention period at 3 months, and (3) the postintervention time point at 6 months or beyond. Interviews were audiotaped, transcribed verbatim, and analyzed using conventional content analysis.

**Results:**

A total of 48 participants (women: 33/48, 69%) completed the interviews, with 24 and 19 completing midpoint and postintervention interviews, respectively. Participants generally had a positive experience in the trial. Many found value in learning about the connection between pain and mental health, considering it an important aspect of their well-being. The health coach played a key role in offering personalized support and guidance. Although the chatbot reinforced pain management strategies, its usefulness and engagement varied based on participants’ prior knowledge of SCD. The pain diary helped increase self-awareness of pain patterns but was perceived as tedious and irrelevant by those without current pain episodes.

**Conclusions:**

This qualitative substudy of the CaRISMA trial showed that participants valued the personalized support of the health coach, education about the connection between stress and pain, and the self-reflection fostered by the pain diary. These findings highlight the potential of digital, patient-centered approaches to address the multifaceted needs of SCD care. For digital interventions, the inclusion of personalized support with ongoing communication appears to be a critical component that can influence treatment adherence and effectiveness.

**Trial Registration:**

ClinicalTrials.gov NCT04419168; https://clinicaltrials.gov/study/NCT04419168

**International Registered Report Identifier (IRRID):**

RR2-10.2196/29014

## Introduction

### Sickle Cell Disease

Sickle cell disease (SCD) is a genetic disorder characterized by recurrent acute episodes of severe pain, commonly referred to as “pain crises,” and chronic pain that can persist between these episodes. The unpredictable nature of SCD-related pain, combined with the physical and emotional burden of living with a chronic illness, leads to high rates of depression, stress, and reduced social support [1,2]. Additionally, stress and depression can further exacerbate pain, creating a cycle in which physical and emotional distress reinforce each other. Historically, pain interventions in SCD have focused on pharmacologic treatments, yet comprehensive biopsychosocial approaches that address both the physical and emotional aspects of SCD pain management remain limited. The 2019 American Society of Hematology guidelines for the management of SCD recommend a comprehensive approach to pain management, including pharmacologic treatments alongside behavioral health interventions that address psychological and social contributors to pain [1]. Despite these recommendations, access to integrated biopsychosocial care remains limited, leaving many individuals with unmet behavioral health and psychosocial coping support needs [3].

### Cognitive Behavioral Therapy for Chronic Pain

Cognitive behavioral therapy (CBT) is a first-line, evidence-based treatment for chronic pain endorsed by the American College of Physicians and the United States Centers for Disease Control and Prevention [4,5]. CBT effectively improves pain outcomes in several chronic conditions, including SCD [6-8], by addressing psychosocial contributors to pain such as maladaptive thought patterns, promoting behavioral activation, and equipping individuals with coping strategies. CBT has also been linked to improved emotional regulation, self-efficacy, and health-related quality of life in patients with chronic pain.

### Digital Interventions for SCD

Despite its effectiveness, access to CBT has been limited by barriers such as lack of transportation, costs, provider shortages, and scheduling conflicts. Digital behavioral interventions offer a promising avenue to expand access to behavioral health support, especially for individuals with chronic illnesses like SCD who face barriers to in-person care; however, the use of digital interventions in SCD has been limited. Digital CBT programs have demonstrated effectiveness for improving pain-related outcomes, coping skills, and mental health across chronic pain populations. Furthermore, when compared with non-CBT mobile apps, CBT-based apps demonstrated greater efficacy for behavior change than non-CBT apps, likely due to structured use of CBT techniques, engagement in cognitive reframing tasks, and empowerment of users [9]. However, there is limited evidence on the effectiveness and user experiences with digital behavioral health programs specifically designed for individuals with SCD [6,10-13], a population with unique medical, cultural, and psychosocial needs. Individuals with SCD report mixed experiences with available digital health interventions: Some found these tools helpful for learning coping strategies and tracking symptoms, while others noted limitations such as insufficient personalization or a lack of cultural relevance to the unique challenges of SCD [14]. In the context of SCD, where pain is deeply intertwined with stress, mood, and self-efficacy, digital CBT offers a targeted and scalable tool for helping individuals manage pain both psychologically and behaviorally, but more high-quality clinical trials of digital CBT interventions are needed.

### Initial Development of a Digital CBT Intervention: Beginning With Addressing the Challenges in SCD Clinical Trials

Clinical trials in SCD face significant recruitment and retention challenges. Insufficient patient participation is a major impediment to the advancement of SCD research, with many studies terminating due to low enrollment [15,16]. A systematic review of 174 SCD clinical trials found that 14% were terminated specifically for failure to enroll participants, with only 35% of completed studies resulting in published manuscripts [17]. These challenges can be particularly pronounced for behavioral interventions, which require consistent engagement and are time-intensive for the participants. Our early research aimed to simultaneously address enrollment challenges and develop an accessible intervention by prioritizing people with lived experience. In collaboration with the SCD community, our group developed a digital CBT app adapted for adults with SCD. To explore the feasibility and preliminary impact of digital behavioral health tools in SCD, our group previously conducted a pilot randomized controlled trial with adolescents and young adults evaluating an earlier version of our CBT app compared with a CBT app for the general population [18]. The study demonstrated improvements in pain, self-efficacy, and depressive symptoms, highlighting the potential for digital interventions to address the biopsychosocial needs of this population. However, about 40% of participants did not engage with the digital interventions, revealing significant barriers to sustained participation.

### Prior Work Refinement and Expansion of the Digital CBT Intervention: Further Addressing Engagement Challenges

To better understand the determinants of digital intervention engagement, we previously conducted a qualitative study using focus groups of adolescents and adults with SCD [18]. Participants identified 5 key engagement factors, including the importance of peer connection, personalized content and coaching, preferences regarding the coach’s background (eg, a coach with lived SCD experience), the value of journaling and symptom tracking, and flexible engagement options [13]. These insights, along with data from our pilot randomized controlled trial, helped inform the design of the Cognitive Behavioral Therapy and Real-Time Pain Management Intervention for Sickle Cell via Mobile Applications (CaRISMA) trial. We randomized participants to 1 of 2 health coach-supported interventions: digital CBT versus digital patient education [19]. Participants also completed pain diaries during the study period. All components were designed to align with the preferences and barriers identified in our prior work, with the goal of creating a flexible, patient-centered digital platform for SCD pain management. Peer support delivered by health coaches was a core component of the intervention, as research suggests that peer-delivered models can build trust, improve engagement, and address barriers to mental health care in historically underserved populations [20-24]. In the primary outcomes study, we found that both the CBT and education interventions led to an improvement in pain interference; however, there was no significant difference between the arms [19].

This study focuses on participants’ experiences in the CaRISMA trial and with the various components of the digital CBT program. Most of the previously published studies on digital interventions focus on single-component interventions [25]. Multicomponent, app-based interventions are less common but emerging [26]. The CaRISMA trial incorporated a multicomponent, culturally tailored platform that included peer-supported digital CBT, symptom tracking, and personalized chatbot engagement. Although there was no difference between groups, we found that digital CBT leads to improved pain-related outcomes [19]. Therefore, a critical next step is to better understand participants’ experiences with the intervention components to in order to refine future approaches and enhance the likelihood of successful implementation in real-world settings.

### Objective of This Study

This qualitative study aimed to gain a deeper understanding of participants’ experiences with the CaRISMA trial, focusing on their perceptions of the health coach, chatbot, and pain diary components. By identifying key factors influencing engagement and satisfaction, we sought to better understand barriers to trial enrollment and retention in the SCD population, evaluate the potential for digital behavioral interventions to address this population’s unique needs, and inform the implementation of culturally appropriate, patient-centered digital health interventions for individuals living with SCD.

## Methods

### Study Design and Setting

The protocol for CaRISMA has been previously published [27]. The CaRISMA trial enrolled adults with SCD who reported chronic pain (ie, pain at least 4 days a week over the past 3 months or longer). Participants of the CaRISMA trial were recruited from 7 comprehensive sickle cell centers and from 4 community-based organizations, either virtually or in person. Participants were randomized 1:1 to either the digital CBT or Education arm. Both arms had access to a health coach and a chat system via a chatbot. All participants were asked to complete daily pain assessments via an electronic pain diary app.

For this qualitative study, we aimed to recruit a diverse group of trial participants, stratifying our sample by depression severity assessed using the 9-item Patient Health Questionnaire (PHQ-9; PHQ-9≥10 vs PHQ-9<10) and trial arm (CBT vs Education). Trial participants were randomly selected for 1-to-1 semistructured interviews at baseline. We sought 24 interview participants from each study arm (CBT or Education), and within each of those 2 sets of 24 participants, we sought 12 participants with high depression severity (PHQ-9≥10) and 12 participants with low depression severity (PHQ-9<10). If a randomly selected participant declined to participate in the baseline interview, another participant was randomly selected until we had achieved a total of 48 interviews. This target was chosen based on a strong likelihood of achieving thematic saturation, the point at which additional interviews are unlikely to provide new insights [28,29]. Participants who completed baseline interviews were invited to participate in follow-up interviews at midtrial (3 months, end of intervention) and post-trial (between 6 months and 12 months) time points.

### Intervention Components

#### Digital CBT

The digital CBT arm included a 12-week, tailored program designed for adults with SCD. It taught participants how to identify negative thoughts and emotions, use cognitive skills and problem-solving techniques, and apply effective coping strategies. The program focused on skill development through practice, homework assignments, and regular check-ins with a health coach.

#### Digital Education

The digital Education arm included comprehensive information about SCD and pain management through an interactive digital platform. Participants learned about chronic pain, lifestyle modifications, and SCD fundamentals while reinforcing their knowledge through interactive assessments. The program encouraged active learning by enabling users to discuss concepts with their health coach.

#### Health Coach

Health coaches—many of whom had personal connections as SCD patients, caregivers, or advocates—offered weekly support through phone calls or text messages, depending on participant preferences. Their primary role was to provide emotional support and encourage active engagement with the digital platform. Each coach underwent comprehensive training and attended weekly group supervision sessions facilitated by a mental health professional and research lead.

#### Chatbot

**Figure 1:**
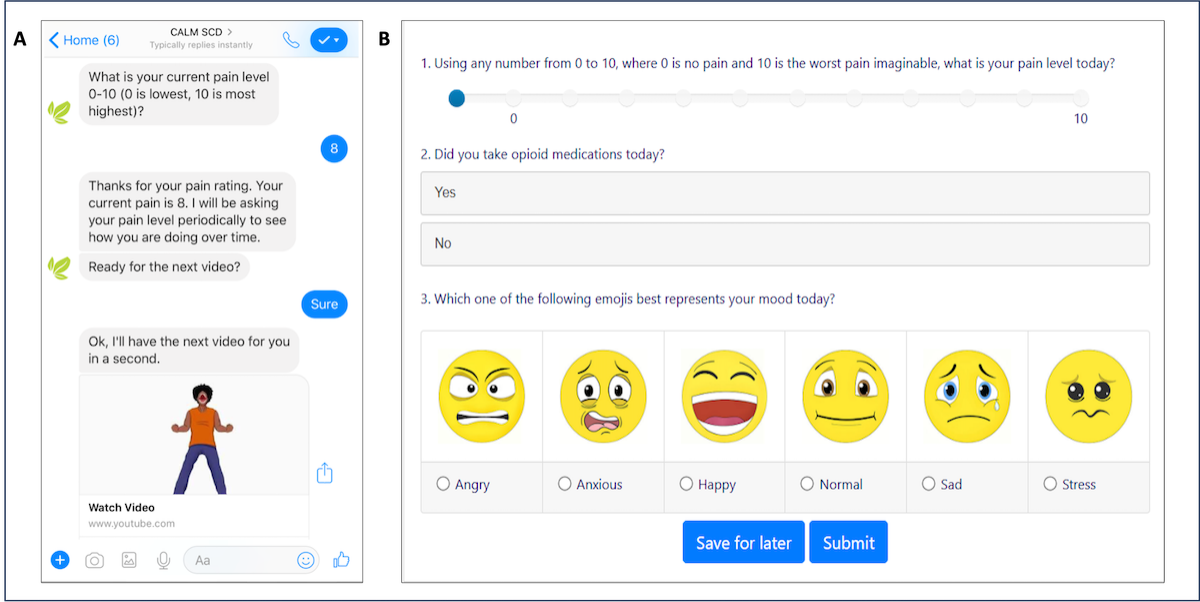
Cognitive Behavioral Therapy and Real-Time Pain Management Intervention for Sickle Cell via Mobile Applications (CaRISMA) components including (A) chatbot interaction and (B) pain diary tracking.

The CaRISMA interventions were delivered by a scripted chatbot (Figure 1), which was developed in collaboration with patients, families, and community partners. The chatbot was accessed through the Facebook Messenger app and offered tailored responses, educational materials, and motivational content in various formats including videos, GIFs, and images, all adapted to participants’ inputs. It also provided round-the-clock conversational support, adapting responses to each participant’s unique circumstances and needs. Users were able to monitor their progress through the program, revisit materials they had completed, and view their accomplishments via progress metrics and achievement rewards.

#### Pain Diary

Patients were asked to track pain levels daily through a mobile web app, as illustrated in Figure 1. They received text reminders during 2-week assessment windows at the start of the study and at 3 months, 6 months, and 12 months. Between these formal tracking periods, they were encouraged to keep logging their pain scores in the app.

### Qualitative Analysis

#### Analytic Approach

We used a qualitative descriptive approach for data collection and analysis, aiming to directly capture and summarize participants’ experiences, thoughts, and feelings related to the interview topics. This approach is frequently used in health and medical research to provide clear and practical insights [28,30]. Here, we sought a holistic understanding of SCD patients’ prior experiences with pain, depression, and treatment for either or both conditions.

Our interview guides for baseline, midpoint, and final interviews (Multimedia Appendix 1) were designed to explore participants’ experiences throughout the trial. Baseline interviews focused on participants’ previous experiences with SCD pain management and their initial impressions of the study. Midpoint interviews gathered feedback on their experiences partway through the trial and any changes they had noticed. The final time point interviews sought information on durable effects and knowledge gained from their trial experiences.

#### Data Collection and Coding Procedures

Data collection and analysis were conducted by skilled qualitative researchers at the Center for Biostatistics and Qualitative Methodology’s Qualitative Core, under the supervision of a qualitative methodologist. Once a randomly selected CaRISMA participant agreed to be interviewed, the interview was scheduled at a time that was convenient for them. To accommodate CaRISMA participants who were located across multiple geographic locations, interviews were conducted telephonically or via teleconferencing software according to the interviewee’s preference. Interviews were audio-recorded and transcribed verbatim, with identifying details redacted. Although we hoped to have one consistent interviewer across the time points, the duration of the study and the logistics resulted in interviews being conducted by 3 interviewers, all of whom received the same training.

Concurrent with data collection, interviewers completed a summary template after each interview to document key topics discussed and capture emerging themes. These summaries allowed the team to assess thematic saturation, which was achieved. A trained qualitative analyst inductively developed an initial codebook based on the content of the interviews. This draft codebook, complete with detailed code definitions, was circulated to the study team to ensure that it reflected relevant topical and theoretical themes important to the study team, as well as the content of the interviews. The codebook was slightly revised to incorporate new codes specific to themes emerging in the midpoint and final interviews. Once codebooks were finalized, qualitative coding was completed with the assistance of MAXQDA 2022 software. For each data set, 10 transcripts were co-coded and then reviewed by 2 independent, trained qualitative coders from the Center for Biostatistics and Qualitative Methodology to ensure quality and consistency in coding. Any coding discrepancies identified were adjudicated by the coders to full agreement. The primary coder completed the coding of the remaining transcripts following the standards established in the codebook and refined through the adjudication process. This coding served as the basis of a conventional content analysis [18], in which the primary coder prepared summaries describing participants’ responses related to each interview guide domain at each time point. These summaries were reviewed by the qualitative methodologist and other study team members to identify the most relevant findings for reporting. Additional manuscripts are planned to present findings related to pain management and depression. This manuscript focuses specifically on participants’ experiences with and perceptions of the CaRISMA trial.

### Ethical Considerations

This study was approved by the University of Pittsburgh Institutional Review Board (STUDY20110346). Informed consent for this study was included in the original consent process for participation in the CaRISMA trial. No compensation was provided for this portion of the study. Participants were compensated for their broader participation in the CaRISMA trial. All study data were stored on secure University of Pittsburgh servers, accessible only to authorized members of the study team. Specifically, access was limited to members of the Qualitative Core who conducted the analysis. Audio files and interview transcripts were labeled with unique study identifiers and did not include participant names or other direct identifiers. All transcripts were de-identified, with personally identifying information redacted during the transcription process

## Results

### Participant Characteristics

A total of 48 participants completed baseline interviews. Of those 48 participants, 24 completed a midpoint interview, and 19 completed a final time point interview following the conclusion of their interactions with CaRISMA. At baseline, the mean age of participants was 36 (SD 9.7) years, and the majority were women (33/48, 69%). Most participants were recruited through clinic visits (38/48, 79%). About two-thirds (33/48, 69%) had attended some college or higher education, while 31% (15/48) had some high school education or a high school diploma or equivalent. One-third of participants (16/48, 33%) were employed, and about one-half of participants (25/48, 52%) reported being on disability. The demographic and clinical characteristics of participants at the baseline, midpoint, and final interview time points are summarized in (Table 1).

Here, we focus on participants’ experiences in the trial, focusing on the unique aspects of the CaRISMA program, including the health coach, chatbot, and pain diary.

**Table 1:** Baseline demographic and clinical characteristics of study participants across time points.

Characteristic		Baseline (N=48)		Midpoint (n=24)		Final (n=19)
Age (years), mean (SD)		36.1 (9.7)		35.7 (10.8)		34.2 (10.8)
**Sex, n (%)**						
	Female	33 (69)	8 (33)		6 (32)	
	Male	15 (31)	16 (67)		13 (68)	
**Race, n (%)**						
	Black/African American	45 (94)	23 (96)		19 (100)	
	Multiple/other/White	3 (6)	1 (4)		0 (0)	
**Education, n (%)**						
	Some high school	3 (6)	1 (4)		1 (5)	
	High school or equivalent	12 (25)	5 (21)		6 (32)	
	Some college	23 (48)	13 (54)		8 (42)	
	Completed college	6 (13)	3 (13)		3 (16)	
	Graduate studies	4 (8)	2 (8)		1 (5)	
**Employment status, n (%)**						
	Employed	16 (33)	7 (29)		8 (42)	
	Unemployed	7 (15)	4 (17)		2 (11)	
	On disability	25 (52)	13 (54)		9 (47)	
PROMIS^a^ pain interference score, mean (SD)		62.2 (7.2)		60.6 (6.9)		60.4 (7.8)
Average pain intensity, mean (SD)		4.0 (2.5)		3.7 (2.3)		3.5 (2.5)
Pain episode frequency, mean (SD)		47.6 (14.3)		48.1 (13.2)		46.5 (13.7)
**ASCQ-Me^b^ measures, mean (SD)**						
	Social functioning impact	46.9 (8.2)	47.5 (8.0)		47.8 (9.8)	
	Emotional impact	48.0 (10.0)	49.3 (9.3)		50.2 (11.0)	
Number of pain diary entries (during the 2-week reminder period), mean (SD)		9 (4)		10 (4)		8 (4)
Depressive symptoms (PHQ-9^c^), mean (SD)		10 (5)		9 (4)		10 (5)
**Depressive symptom severity, n (%)**						
	Minimal (0-4)	5 (12)	4 (19)		2 (13)	
	Mild (5-9)	17 (40)	8 (38)		7 (44)	
	Moderate (10-14)	8 (19)	5 (24)		3 (19)	
	Moderately severe (15-19)	12 (28)	4 (19)		4 (25)	
	Severe (20-27)	1 (2)	0 (0)		0 (0)	
Brief PCS^d^, mean (SD)		9 (4)		9 (4)		8 (4)
GAD-7^e^, mean (SD)		9 (5)		7 (4)		8 (5)
SCSES^f^, mean (SD)		29 (6)		29 (5)		29 (6)

^a^PROMIS: Patient-Reported Outcomes Measurement Information System.

^b^ASCQ-Me: Adult Sickle Cell Quality of Life Measurement Information System.

^c^PHQ-9: 9-item Patient Health Questionnaire.

^d^PCS: Pain Catastrophizing Scale.

^e^GAD-7: 7-item Generalized Anxiety Disorder.

^f^SCSES: Sickle Cell Self-Efficacy Scale.

### Overall Trial Experience

Most participants reported a positive overall experience with the CaRISMA study, emphasizing that it provided a “safe space” to learn and express themselves without fear of judgment. One participant noted:

I enjoy doing it. I really enjoyed—like I said, I learned a lot. And I like the fact that I can say how I feel in a safe, safe space. [...] the thing I think I appreciated the most about the study was, no matter what I say, good, bad, or indifferent, it is appreciated, and I felt safe. I can say it exactly how I feel.

P31

Others appreciated the study’s focus on the connection between chronic illness and mental health, noting that such an approach was rare but much needed. One participant noted:

I feel like the study went well. [...] The fact that you guys thought of doing something like this to think on mental health and just treating symptoms differently, I felt like was great for the community because a lot of us did not know anything about CBT.

P68

Additionally, several participants found the exploration of the link between pain and depression particularly valuable, highlighting the need for greater awareness of this relationship within the sickle cell community. One participant noted:

Yeah, it was good overall. I think that I was quite impressed that there's something being studied about, you know, like taking care of the relationship between chronic illness and mental health. I hadn’t seen that before so that was a good one for me. Yeah, so it was good generally.

P107

Despite these positive aspects, participants also expressed a desire for more follow-up and reminders throughout the study. Many felt that regular check-ins and clearer communication could have improved their engagement and overall experience:

Yeah, I feel like that’s, that’s, that would help the study be so much more successful or everybody's feeling on the same page, just because — I mean, not saying that it's not successful now, but I feel like, you know, if you have people checking me in frequently and letting you know, hey, you know, you had this option, this option, this option, you maybe should try it out, see if this helps, whatever. It was just it was a lack of communication.

P104

Participants expressed mixed preferences regarding in-person versus social media–based engagement, with some valuing the sense of community and real-time connection that in-person meetings offer, while others appreciated the convenience and accessibility of digital platforms. One participant emphasized the benefits of in-person interactions, stating “It would be nice to be in-person, you know, we all get together now” (P110). Another noted the challenges of an in-person engagement:

Oh, I like in-person. Um, the only thing is, you know, distance, transportation, things like that, schedules of people that are working, you know. But in-person is always a good one, because you, for me, the most important thing about any person is a sense of community, um, and connection to someone else in real-time, right. But other than that, I think on, like, a walk through a study, because, you know, scheduling conflict, and time and transport and all those things might make in-person maybe a bit more difficult.

P107

Participants also had mixed opinions on the most effective social media platform for engagement, with some favoring Snapchat due to its artificial intelligence chatbot feature, which they believed could enhance interactions and provide a more conversational experience. Others preferred Instagram, citing its widespread use and strong messaging capabilities, suggesting that a more active presence with regular updates and resources could increase engagement. Another participant recommended a separate app for the study. If a separate app is not feasible, then Instagram is the next best option:

Yeah, I, I think, I think the app will be the best route. But if, if you couldn't, if, if it was added on other social media platform, I feel like Instagram will be a real, a real good one, because I think Instagram is probably more widely used, especially when it comes to, like, messaging. Um, so I, I think it will be, yeah, I think you'll get more of a reach on Instagram. Because I've been on CaRISMA Instagram page, and, I mean, it’s, it's okay, but it don't, it don't really have [...] life on it. You know, that, that will be the only thing that they will have to do is just bring more life to the page.

P68

Others saw value in leveraging existing platforms to reach a broader audience. Overall, participants highlighted the importance of selecting a platform that aligns with user preferences while ensuring consistent engagement and accessibility.

### Health Coach Interactions

Participants appreciated that the health coach had SCD *or* knew someone (eg, a family member) who has SCD. Participants thus felt understood by the health coach. For instance, one participant said, “I just like to be able to talk to my coach, because she’s going through what I’m going through. So, it’s, it’s good to be able to talk to somebody” (P30). Another participant similarly said, “I loved it [the health coach]. It was like, you know, talking to somebody that I can, you know, relate to. Um, that was, that was a great thing. You know? Easy to communicate. You know” (P40).

Talking to someone who was knowledgeable proved to be very helpful. This led to enduring effects beyond the health coach interactions, with participants noting that they learned the importance of talking and reaching out to someone when they are upset or in pain. In at least one case, a participant sought counseling after completion of the CaRISMA program, as a result of learning how helpful it could be to talk to someone:

There is one thing that we did choose to implement, which was, you know, just kind of self-care and mental health check-ins, and that’s something that on the side of after speaking with my CaRISMA coach about that, that’s something that I decided to sign myself up for. So, I signed up for counseling through my church and we have little weekly check-ins; so that’s been very helpful. [...] So yes, I, I would, I would think that, that was definitely was something that, that’s helpful in the event that there is a depression or anxiety episode that rises.

P53

Participants also appreciated that the health coach was willing to be flexible when scheduling meetings, as the health coach understood the unpredictability of SCD. For instance, one participant said,

I feel like she’s [...] very helpful. She’s, uh, very, like, mindful of my time and my, my situation, like she understands that there are some days where I could just really just be ready to collab and have a long conversation. And then there are other days where I may not feel well and I’m not interested in really just talking on the phone, especially if I’m in the hospital or at home trying to relax. So, that’s why I like; she comes in and texts and if, if we scheduled an appointment and that’s not good day, she’s very flexible. So, uh, I kinda like how everything is going with, with the coach that I have.

P53

Some participants lamented limited interactions with their health coach, wishing that they had more time to get to know them. For example, one participant said, “I kinda wish I had the, more time to talk to her more, so we could kinda get like a better understanding of each other” (P67). The participant added that, ideally, they would like to speak to the health coach “every week because with sickle cell stuff can change so quickly, so you never know what I could be doing this week or what could happen next week. So, I would say, like, every week but I know, like, schedules and work and school and different stuff puts a burden on me trying to check in with her every week. So, I mean, text messages is fine, but conversation is different than texting, so, that’s what I mean” (P67).

### Chatbot Use and Feedback

Most participants found the chatbot to be helpful, barring technical difficulties. Participants primarily enjoyed the chatbot if they gained new insights from it. Some participants said that the information they learned helped to reframe the way they think about their SCD. One participant found this to be an enlightening experience:

I actually learned something new. I didn’t think I was going to learn anything. [...] So we know that the sickle cells are crescent shaped. [...] Well, I learned that different types of, you know, genotypes sometimes the sickle cells are shaped differently. Because I have [inaudible] mine are more shaped like crystals instead of crescents. So that was new. I was like what! And I love crystals [laugh]. You know, they kind of made me like the sickle cell a little bit. Like okay, girl, we’re twins! [...] I really did not know that. I was like oh my God I’ve had sickle cell for 34 years and I did not know that.

P82

Some participants found the chatbot helpful for reinforcing pain management strategies, while others felt it provided limited new information for those already knowledgeable about their condition. For participants who gained new insights, the chatbot was a valuable learning tool.

Some participants also noted that the insight they gained challenged certain “preconceived notions” they held about SCD:

Um, yeah, there’s been...um, information that I...probably didn’t think about...before. On that. And, that you had, um — you know, it’s just like you think this is logically, this is certain information you had in your head about stuff, and you find out that, oh, my god, that they makes sense, this, this — do you understand what I mean? [...] Okay. So, if I had a preconceived notion on a particular aspect of sickle cell; I don’t know, if for any reason like…it could be like an old wives’ tale. And then, on looking, going through that I get a different information and I’m like, oh, that makes sense; why did I ever think this is what it was? Yeah, that’s what I mean.

P69

In contrast, participants who did not find the chatbot to be helpful reported that it did not teach them anything new. The participant explained that the chatbot was more useful for “people who [...] don’t know much about their own disease”:

To be honest, I think it was more unhelpful to me, because the questions that it asked are, are questions that I already know the answer to. Does that make sense? Like, um, some people don't know what kind of sickle cell they have, or they don't know their triggers or, or things like that. And my mom taught us growing up, like, to be able to speak for yourself when you went to the doctor, so I know what kind of sickle cell I have. I know — you know what I’m saying — my triggers, I know, um, my medications and the dosages and stuff like that. So, it would be helpful for some people who aren't really, I guess who don't know much about their own disease. Does that make sense? But I...I think I know my disease and my body pretty well to where that was, it wasn't helpful to me.

P80

Frequently, participants who did not use the chatbot suggested that it should take a more proactive role in encouraging participants to interact with it, rather than relying on users to engage on their own. For example, when asked what would improve their use of the chatbot, one participant asked for a reminder: “Train the bot to just kind of reach out and be, like, ‘Hey, you haven’t answered the question or asked a question in a couple days; is everything good or would you like to resume’-type thing” (P39). The need for reminders was particularly salient for anyone who did not regularly interact with Facebook Messenger outside of the study and might therefore not see it in the course of their day.

### Use of the Pain Diary

One participant added that filling out the pain diary—and being able to show the pain diary to their health care providers—was very helpful:

And also, to see that part in the, in the study where they was asking, like, how you feel, like that was, that’s something I wish was everywhere, honestly; I wish I had that everywhere. I wish you could, like, show the doctors how you feel and whatnot, because even if they did put it everywhere, I feel like if it was standardized, they probably would know how to deal with different pain levels, you know, and, and that would probably be able to help them structure, um, medication doses. Like I feel like that would be so helpful cuz they can see it, and it's not more of you trying to struggle and explain it to them; they already see it; they know exactly how this is affecting you. This is, this is what works for them, many other warriors on this level; let's give them the same — and I'm telling you, I feel like it would be so helpful. I feel like that needs to be implemented in every hospital for every, every person just — or not anybody. Um, but yeah, I, I, I think that was probably the, the biggest thing for me.

P68

Some participants found completing the pain diary daily redundant or tedious, particularly on pain-free days, leading them to develop their own strategies to manage this task. One noted:

To me personally because it would come every day. I would get the email for it every day. And at a certain point it felt redundant if I wasn’t in pain for a few days or weeks at a time. Having to fill it out every day. So it got to the point where if I had pain I would say okay yeah I’ll go fill it out now. Instead of every day putting no pain.

P115

Some participants appreciated completing the pain diary daily, even on pain-free days, as it allowed them to track their symptoms and emotions. It also helped them reframe their experience, reminding them that not all days are “bad” and that pain episodes eventually subside. For example, one participant stated:

I mean, I like the pain diary because then it shows me, like, you know, what I write. Pretty much write down everything on my own. To do it every day just to know okay I’m feeling bad this day but this day I was okay. So that kind of helps.

P104

Participants also appreciated having a record of their pain and pain medication usage. As one participant described it:

Not that I enjoy, but I do look forward to the daily pain diary; it just, it does make me stop and think about, you know, what pain I’m in and have I taken meds and do I, you know, what can I do to not decrease but to minimize the pain and, in turn, minimize, I guess, the amount of medication. So that is helpful.

P58

Thus, participants felt that the pain diary helped to increase their awareness of their pain and feelings, helping them make more informed decisions about their pain management strategies.

Representative quotes are summarized by intervention component in Table S1 in Multimedia Appendix 2.

## Discussion

### Principal Findings

Digital health interventions have emerged as a promising tool to enhance chronic pain management and coping. Based on feedback from people with lived experience, we designed the CaRISMA trial to equip adults with SCD with the tools, resources, and support systems necessary to improve long-term chronic pain outcomes. In the primary CaRISMA trial, we found that both digital interventions (CBT and Education) resulted in improvements in the primary pain outcome, pain interference, at 6 months [19]. Additionally, greater engagement with health coaches was associated with greater improvements in pain interference. The qualitative study reported here enhances our understanding of the first peer-supported digital CBT intervention for adults with SCD by examining participant experiences with its key components: the health coach, chatbot, and pain diary.

### Overall Experience

Overall, participants reported a positive experience with the CaRISMA trial, appreciating the safe space provided for open discussions and the opportunity to learn about the connection between pain and mental health. Although some participants preferred face-to-face interactions, logistical barriers such as transportation and health limitations reinforced the value of flexible digital formats, consistent with findings in other chronic disease populations [31,32]. Regarding the delivery platform, some participants valued engagement via social media (eg, Facebook, Instagram), appreciating its convenience, accessibility, and ability to provide on-demand support. Participants also had varying opinions on which social media platform would be most effective, with some favoring Snapchat due to its chatbot feature, while others preferred Instagram for its broad user base and messaging capabilities. Given the evolving nature of social media, leveraging multiple digital platforms may enhance engagement by catering to diverse communication preferences. Alternatively, considering the shifting landscape of social media usage, consolidating resources into a single, dedicated app, separate from social media, could provide a centralized and streamlined user experience. These findings also highlight the importance of offering flexible, accessible digital health interventions that accommodate the unique needs, preferences, and logistical challenges faced by individuals living with chronic conditions like SCD.

### Health Coach Experience

Participants strongly valued their health coach’s lived experience with SCD, highlighting the need for culturally concordant and condition-specific health coaching. In other chronic disease populations, tailored peer support has contributed to enhanced trust, self-efficacy, satisfaction, engagement, and adherence by offering emotional support, shared experiences, and practical guidance [20,21,33]. In the CaRISMA trial, many participants appreciated that their health coach had SCD or personal connections to someone with SCD, which contributed to feeling understood and supported. Some participants also reported that interactions with their health coach motivated them to seek mental health counseling, demonstrating the intervention’s potential for long-lasting impact that possibly extends beyond pain management.

Participants found the health coach relationship so valuable that many expressed a desire for more frequent contact. We designed the CaRISMA program to consist of weekly check-ins; however, the unpredictability of pain episodes for both participants and health coaches interfered with check-ins. Our findings are aligned with those of previous studies demonstrating that more frequent, flexible check-ins improve engagement and adherence in chronic disease populations [34,35]. To optimize future interventions, health coach check-ins should be frequent and adaptable, incorporating scheduled and on-demand support. A multimodal approach, incorporating text messages, phone calls, and video check-ins, could help meet individual needs.

### Chatbot Experience

The chatbot received mixed responses; some participants found it helpful for reinforcing pain management strategies, while others felt it provided limited new information. These differences may reflect variation in prior knowledge, learning preferences, higher health literacy, or the degree to which participants engaged with other components of the intervention. Digital interventions incorporating chatbots have been recognized as an effective way to deliver behavioral coaching, track symptoms, and strengthen patient engagement [36]. Although some participants had a positive or eye-opening experience with the feature, others suggested that the chatbot should be more proactive, initiating conversations and providing reminders rather than waiting for users to engage. Previous research highlights that adaptive, artificial intelligence–driven chatbots that tailor responses based on user engagement and knowledge levels improve overall effectiveness in digital health interventions [37]. This approach could be incorporated into future digital interventions for adults with SCD.

### Pain Diary Experience

The pain diary helped some participants track pain trends, but others found it redundant and unnecessary on pain-free days. Daily pain diaries are widely used in both SCD and broader chronic pain populations to monitor pain patterns and inform treatment strategies. In previous studies, patient experiences with these diaries have varied, reflecting both benefits and challenges. For instance, in the Pain in Sickle Cell Epidemiology Study (PiSCES), 60% of participants submitted fewer than 5 months of the expected 6 months of pain diaries, indicating challenges in sustained daily reporting [38]. In the CaRISMA trial, some participants mentioned that tracking their pain helped them gain perspective on their condition, allowing them to recognize that not every day was a “bad” day. This is a sentiment echoed in prior research on digital self-monitoring tools for chronic pain [14,39]. However, others recommended the use of the pain diary only when participants are experiencing pain. Research has shown that user fatigue is a common barrier to adherence in digital health interventions, particularly when data entry is required daily without immediate benefits [14,40,41]. To enhance usability, customizable pain tracking options allowing participants to self-select tracking frequency might be helpful. Additionally, integrating mood tracking and other symptom assessments alongside the pain diary may provide a more holistic view of well-being.

### Comparison With Prior Work and Future Directions

The CaRISMA trial offered a novel approach to pain management in SCD by integrating a culturally adapted, multicomponent digital intervention within a historically marginalized and medically underserved population. Our focus on a historically marginalized group with specific cultural needs contributes new evidence on tailoring digital behavioral interventions for high-need populations. Unlike previous digital health trials that often focus on single-component interventions, the CaRISMA trial evaluated a comprehensive model combining digital CBT, peer support via health coaches, symptom monitoring, and chatbot technology. This study also highlights the importance of tailoring digital behavioral health interventions to the unique needs of individuals with SCD. The findings suggest that a multicomponent approach can enhance engagement and effectiveness. Future trials should consider the following recommendations: (1) enhanced health coaching that involves frequent and flexible coach check-ins, including on-demand support, to improve participant engagement; (2) an adaptive and interactive chatbot that offers personalized education based on an individuals’ knowledge, needs, and preferences (eg, chatbot with adjusted tone and content based on patient-reported depression and pain severity or providing layered content depending on the user’s baseline familiarity with SCD education and management); and (3) flexible and customizable self-monitoring tools, such as pain diaries that allow users to adjust tracking frequency, skip entries on low-pain days, or annotate with additional symptoms and reflections, as well as view visual summaries of pain trends to support provider communication and self-awareness. Future research should also develop and test digital interventions that incorporate other therapeutic approaches, such as acceptance and commitment therapy [42,43] and mindfulness-based stress reduction [44,45]. Digital interventions may also offer a form of cognitive escapism, a concept referring to psychological relief and distraction from distressing symptoms. For instance, Anto et al [46] found that digital tools can help users disengage from pain and emotional burdens through structured, engaging content. Within the context of SCD, a digital CBT program may provide both therapeutic and escapist benefits, helping individuals better manage their symptoms while finding temporary relief from the psychological weight of chronic illness, which may be relevant to coping and engagement. These processes should be further investigated in adults with SCD.

### Strengths and Limitations

This study has several strengths. First, to our knowledge, this is the first qualitative study to explore patient experiences with a peer-supported digital CBT intervention tailored for chronic pain in adults with SCD. Second, it fills a gap in understanding SCD patients’ perspectives on digital pain tools by examining multiple intervention components (health coach, chatbot, and mobile pain diary) and how each contributed to engagement and self-management. Third, by evaluating participants’ willingness and ability to use a mobile pain-tracking platform in a real-world setting, the study provides valuable insights for the design and implementation of future digital health interventions. Finally, this study captured real-world barriers and facilitators to digital health engagement in a medically underserved, high-burden population with specific, actionable feedback regarding reminder frequency, chatbot utility, and diary fatigue that can inform digital health optimization.

However, the study also has limitations. First, this study does not account for participants’ underlying mental health conditions, which may have influenced their responses to the interventions. Second, although participants were randomly selected for interviews, selection bias remains possible, as only 48 of the 115 individuals contacted agreed to participate. Third, attrition across interview time points may have introduced bias, as participants who remained engaged may differ systematically from those who dropped out. Although no clear patterns were observed among nonresponders, those who disengaged may differ in unmeasured ways from those who completed all interviews. This may reflect health issues, competing demands, limited perceived benefit, logistical barriers, or stronger motivation to remain involved. It is also possible that retention was skewed toward individuals with particularly strong feelings about the intervention or more positive experiences with the interventions who were more motivated to remain engaged. Although much of our feedback was positive, it is possible that more critical voices did not engage, although we have no data to support this speculation. Last, most participants had at least some college education, which may limit generalizability to individuals with lower educational attainment. As participants were also recruited from specialty centers and community-based organizations, findings may not reflect the broader SCD population

### Conclusions

Overall, participants found the CaRISMA program to be a supportive space for learning, self-reflection, and symptom management. Digital interventions offer a scalable and accessible approach to addressing pain care disparities within the underserved SCD population. Regular, proactive communication emerged as essential for participant engagement, with inconsistent interaction likely contributing to dropout rates in this and similar studies. Future digital interventions should prioritize regular communication among participants, health coaches, and research teams to enhance retention and overall effectiveness. Additional research should focus on developing culturally appropriate, patient-centered, and adaptable interventions that address the diverse needs within the SCD population.

## Appendix

Multimedia Appendix 1 Interview guides.

Multimedia Appendix 2 Representative Quotes by Intervention Component (Health Coach, Chatbot, and Pain Diary).

## Abbreviations

CaRISMA: Cognitive Behavioral Therapy and Real-Time Pain Management Intervention for Sickle Cell via Mobile Applications
CBT: cognitive behavioral therapy
PHQ: Patient Health Questionnaire
PiSCES: Priapism in Sickle Cell Study
SCD: sickle cell disease

## Data Availability

Due to the sensitive nature of the data, the data sets generated and analyzed during this study are not publicly available. However, de-identified numeric data are available from the corresponding author upon reasonable request.
