# Supplementary material for: Experiences With a Multicomponent Digital Behavioral Pain Management Intervention for Adults With Sickle Cell Disease: Qualitative Analysis of the CaRISMA Trial
Source: JMIR Hum Factors. 2025 Aug 5;12:e73719. doi: 10.2196/73719 (PMC12365560; doi:10.2196/73719)
Supplement: Multimedia Appendix 1 [file humanfactors_v12i1e73719_app1.docx]

**Multimedia Appendix 1**.

*Semi-Structured Interview Guides*

Baseline Interview guide

Today, we’ll be talking about your experiences with sickle cell anemia, pain, depression, and treatment for those conditions. I’m going to ask about each of those individually, and then we’ll have some questions about this trial specifically.

To begin with, tell me about what it has been like living with sickle cell anemia. You can tell me about this in any way that you would like.

Now, tell me about the pain that you have experienced with SCA.

How does your pain affect your life?

What treatments have you used to treat your pain?

Tell me about your experiences using narcotics to treat your pain.

- - How easy or difficult is it to access narcotics to treat your pain?
  - Do you experience any stigma from healthcare providers or from friends or family
    as a result of using narcotics for your pain?
  - Is there anything that you don’t like about using narcotics to treat your pain?
  - Is there anything about using narcotics to treat your pain that you think is
    valuable?
  - How do you think that the opioid epidemic in this country has influenced your
    ability to access narcotics for your pain?

Are there any other ways that you cope with your pain?

Now, I’d like to talk about your experiences with depression. Would you describe yourself as depressed? Why or why not?

If you’ve been depressed, how has that affected your life?

How has it affected the pain that you experience from your SCA? Do you think
 that the pain and depression affect each other at all?

Has your depression ever affected your treatment for SCA? What about your
pain from SCA?

Have you ever had any behavioral treatments for any condition – i.e., depression, for another issue or condition, or for your pain? By behavioral treatments, I mean anything that isn’t a medication, like talk therapy.

If yes: How did you feel about that treatment? Was it helpful?

For everyone: What do you think about behavioral treatments in general?

As part of this study, you’ll receive a form of behavioral treatment for your SCA pain, delivered to you via a smartphone. What are your thoughts about participating in behavioral treatment as part of this study? What are your thoughts about participating in it through a smartphone?

Have you had any interactions with your health coach yet? If so, tell me what those interactions have been like?

What, if anything, has been helpful about talking with them?

Is there anything that you don’t like or would change about your interactions with
your health coach?

Lastly, I’d like to ask about your decision to take part in this study. What made you want to participate? What are your hopes about what might happen as a result of being part of this study?

Have you ever participated in a study related to SCA before? If so, what was that like? Based on that experience, is there anything you think we should do, or should avoid, as part of this study?

That was my last question. Is there anything else that you think we should know?

3-month interview guide

First, tell me what you thought of the CaRISMA trial in general.

Do you feel that participating was helpful to you?

Did you have any problems at part of the trial?

Tell me about your interactions with your health coach?

What, if anything, was helpful about talking with your health coach?

What, if anything, did you not like or would you change about those interactions?

Now, I’d like to ask about the treatment you received. It is my understanding that you received (CBT/m-Education).

Tell me about your experiences with the CBT/m-Education?

- - What did you think about the treatment in general?
  - How did you feel about doing it on your phone?
  - Has it helped you to manage your pain?
  - What about your depression?
  - Was there anything that wasn’t helpful, or that you didn’t like?
  - Can you tell me one strategy or tip that you learned from it, that you’ve used?

Are there any strategies or tips that you have not used? Is there any reason you didn’t use it?

Was there anything about CBT/m-Education that surprised you, or that you weren’t expecting when you started?

Do you think any differently about behavioral treatments like CBT/m-Education than you did when you started?

Do you think that other people with SCA should receive CBT/m-Education to help them with their pain? Why or why not?

Next, I’d like to ask you about your experiences with SCA-related pain since starting this study. How has your level of pain and how you feel about it been in the last few months?

Have you had any pain crises since starting the study?

Did you deal with that any differently than you have in the past?

Have you used any narcotics to manage your pain in the last few months?

Was that experience any different than it was before participating in the study?

What, if anything, has motivated you to keep participating in this study?

Based on this experience, would you be willing participate in research again?

Can you tell me more about why or why not?

What, if anything, do you think this study has done well?

Is there anything the study could have done differently to improve your experience?

Are there any questions that you have about your SCA, or pain and pain treatment, that you wish we had better answers to?

That was my last question. Is there anything else that you think we should know?

Final (6-12 month) interview guide

Today, we’re going to be talking about how you’ve continued to use what you learned in CaRISMA (or how and why you haven’t), as well as how you are doing today.

You’ve now been done with the CaRISMA trial for (time period). As a reminder, CaRISMA was the study where you received CBT/m-Education, and had a health coach, to help you with your SCA pain.

I’d like to ask you about your experiences with SCA-related pain since you completed the study. How has your level of pain been in the last few months?

How have you been dealing with your pain during this time period?

Have you had any pain crises since you stopped participating in the study?

If yes, how did you deal with that crises?

Did you deal with that any differently than you have in the past?

Have you used any narcotics to manage your pain in the last few months?

Was that experience any different than it was before participating in the study?

Have you been depressed at all since leaving the study? If so, tell me about what that has been like for you.

Have you used any of the strategies that you learned in CBT/m-Education to manage your pain since leaving the study? (if no, skip to next question)

If so, what strategies did you use?

What about to manage your depression? If so, what strategies did you use?

Have you used anything else that you might have learned from your health coach to manage either depression or pain since stopping the study? If so, what did you use? (if no, skip to next question)

Are there any strategies you have *stopped* using since leaving the study? If so, what are they? When did you stop using them? Are there any reasons you stopped using them?

Is there anything that you think this study did particularly well?

What, if anything, do you think that you think this study should have done differently?

Are there any questions that you have about your SCA, or pain and pain treatment, that you wish we had better answers to?

That was my last question. Is there anything else that you think we should know?
