# Supplementary material for: Experiences With a Multicomponent Digital Behavioral Pain Management Intervention for Adults With Sickle Cell Disease: Qualitative Analysis of the CaRISMA Trial
Source: JMIR Hum Factors. 2025 Aug 5;12:e73719. doi: 10.2196/73719 (PMC12365560; doi:10.2196/73719)
Supplement: Multimedia Appendix 2 [file humanfactors_v12i1e73719_app2.docx]

| **Theme/**  **App component** | **Participant ID** | **Representative Quote** |
| --- | --- | --- |
| **Overall Trial Experience** |  |  |
|  | 107 | “Yeah, it was good overall. I think that I was quite impressed that there's something being studied about, you know, like taking care of the relationship between chronic illness and mental health. I hadn’t seen that before so that was a good one for me. Yeah, so it was good generally.” |
|  | 31 | “I enjoy doing it. I really enjoyed — like I said, I learned a lot. And I like the fact that I can say how I feel in a safe, safe space. […] the thing I think I appreciated the most about the study was, no matter what I say, good, bad, or indifferent, it is appreciated, and I felt safe. I can say it exactly how I feel.” |
|  | 68 | “I feel like the study went well. […] The fact that you guys thought of doing something like this to think on mental health and just treating symptoms differently, I felt like was great for the community because a lot of us did not know anything about CBT.” |
|  | 77 | “Yeah, it was good overall. I think that I was quite impressed that there's something being studied about, you know, like taking care of the relationship between chronic illness and mental health. I hadn’t seen that before so that was a good one for me. Yeah, so it was good generally.” |
|  | 104 | “Yeah, I feel like that's, that's, that would help the study be so much more successful or everybody's feeling on the same page, just because — I mean, not saying that it's not successful now, but I feel like, you know, if you have people checking me in frequently and letting you know, hey, you know, you had this option, this option, this option, you maybe should try it out, see if this helps, whatever. It was just it was a lack of communication.” |
|  | 110 | “It would be nice to be in-person, you know, we all get together now.” |
|  | 107 | “Oh, I like in-person. Um, the only thing is, you know, distance, transportation, things like that, schedules of people that are working, you know. But in-person is always a good one, because you, for me, the most important thing about any person is a sense of community, um, and connection to someone else in real-time, right. But other than that, I think on, like, a walk through a study, because, you know, scheduling conflict, and time and transport and all those things might make in-person maybe a bit more difficult.” |
|  | 68 | “Yeah, I, I think, I think the app will be the best route. But if, if you couldn't, if, if it was added on other social media platform, I feel like Instagram will be a real, a real good one, because I think Instagram is probably more widely used, especially when it comes to, like, messaging. Um, so I, I think it will be, yeah, I think you'll get more of a reach on Instagram. Because I've been on CaRISMA Instagram page, and, I mean, it’s, it's okay, but it don't, it don't really have [inaudible 0:37:19.1] life on it. You know, that, that will be the only thing that they will have to do is just bring more life to the page.” |
| **Health Coach Experience** |  |  |
|  | 30 | “I just like to be able to talk to my coach, because she’s going through what I’m going through. So, it’s, it’s good to be able to talk to somebody.” |
|  | 40 | “I loved it [the health coach]. It was like, you know, talking to somebody that I can, you know, relate to. Um, that was, that was a great thing. You know? Easy to communicate. You know.” |
|  | 53 | “There is one thing that we did choose to implement, which was, you know, just kind of self-care and mental health check-ins, and that’s something that on the side of after speaking with my CaRISMA coach about that, that’s something that I decided to sign myself up for. So, I signed up for counseling through my church and we have little weekly check-ins; so that’s been very helpful. […] So yes, I, I would, I would think that, that was definitely was something that, that’s helpful in the event that there is a depression or anxiety episode that rises.” |
|  | 53 | “I feel like she’s […] very helpful. She’s, uh, very, like, mindful of my time and my, my situation, like she understands that there are some days where I could just really just be ready to collab and have a long conversation. And then there are other days where I may not feel well and I’m not interested in really just talking on the phone, especially if I’m in the hospital or at home trying to relax. So, that’s why I like; she comes in and texts and if, if we scheduled an appointment and that’s not good day, she’s very flexible. So, uh, I kinda like how everything is going with, with the coach that I have.” |
|  | 30 | “I just like to be able to talk to my coach because she’s going through what I’m going through. So, it’s good to be able to talk to somebody.” |
|  | 67 | “I kinda wish I had the, more time to talk to her more, so we could kinda get like a better understanding of each other.” |
|  | 67 | “…every week because with sickle cell stuff can change so quickly, so you never know what I could be doing this week or what could happen next week. So, I would say, like, every week but I know, like, schedules and work and school and different stuff puts a burden on me trying to check in with her every week So, I mean, text messages is fine, but conversation is different than texting, so, that’s what I mean.” |
| **Chatbot Experience** |  |  |
|  | 82 | “I actually learned something new. I didn’t think I was going to learn anything. […] So we know that the sickle cells are crescent shaped. […] Well, I learned that different types of, you know, genotypes sometimes the sickle cells are shaped differently. Because I have [inaudible] mine are more shaped like crystals instead of crescents. So that was new. I was like what! And I love crystals [laugh]. You know, they kind of made me like the sickle cell a little bit. Like okay, girl, we’re twins! […] I really did not know that. I was like oh my God I’ve had sickle cell for 34 years and I did not know that.” |
|  | 69 | “Um, yeah, there’s been…um, information that I…probably didn’t think about…before. On that. And, that you had, um — you know, it’s just like you think this is logically, this is certain information you had in your head about stuff, and you find out that, oh, my god, that they makes sense, this, this — do you understand what I mean? […] Okay. So, if I had a preconceived notion on a particular aspect of sickle cell; I don’t know, if for any reason like…it could be like an old wives’ tale. And then, on looking, going through that I get a different information and I’m like, oh, that makes sense; why did I ever think this is what it was? Yeah, that’s what I mean.” |
|  | 80 | “To be honest, I think it was more unhelpful to me, because the questions that it asked are, are questions that I already know the answer to. Does that make sense? Like, um, some people don't know what kind of sickle cell they have, or they don't know their triggers or, or things like that. And my mom taught us growing up, like, to be able to speak for yourself when you went to the doctor, so I know what kind of sickle cell I have. I know — you know what I’m saying — my triggers, I know, um, my medications and the dosages and stuff like that. So, it would be helpful for some people who aren't really, I guess who don't know much about their own disease. Does that make sense? But I…I think I know my disease and my body pretty well to where that was, it wasn't helpful to me.” |
|  | 39 | “Train the bot to just kind of reach out and be, like, ‘Hey, you haven’t answered the question or asked a question in a couple days; is everything good or would you like to resume’-type thing.” |
| **Pain Diary Experience** |  |  |
|  | 68 | “And also, to see that part in the, in the study where they was asking, like, how you feel, like that was, that’s something I wish was everywhere, honestly; I wish I had that everywhere. I wish you could, like, show the doctors how you feel and whatnot, because even if they did put it everywhere, I feel like if it was standardized, they probably would know how to deal with different pain levels, you know, and, and that would probably be able to help them structure, um, medication doses. Like I feel like that would be so helpful cuz they can see it, and it's not more of you trying to struggle and explain it to them; they already see it; they know exactly how this is affecting you. This is, this is what works for them, many other warriors on this level; let's give them the same — and I'm telling you, I feel like it would be so helpful. I feel like that needs to be implemented in every hospital for every, every person just — or not anybody. Um, but yeah, I, I, I think that was probably the, the biggest thing for me.” |
|  | 115 | “To me personally because it would come every day. I would get the email for it every day. And at a certain point it felt redundant if I wasn’t in pain for a few days or weeks at a time. Having to fill it out every day. So it got to the point where if I had pain I would say okay yeah I’ll go fill it out now. Instead of every day putting no pain.” |
|  | 104 | “I mean, I like the pain diary because then it shows me, like, you know, what I write. Pretty much write down everything on my own. To do it every day just to know okay I’m feeling bad this day but this day I was okay. So that kind of helps.” |
|  | 58 | “Not that I enjoy, but I do look forward to the daily pain diary; it just, it does make me stop and think about, you know, what pain I’m in and have I taken meds and do I, you know, what can I do to not decrease but to minimize the pain and, in turn, minimize, I guess, the amount of medication. So that is helpful.” |
